# COVID‐19 after BNT162b2 two‐dose primary series does not improve the efficacy of a booster dose in nursing home residents

**DOI:** 10.1002/clt2.12224

**Published:** 2023-03-22

**Authors:** Hubert Blain, Edouard Tuaillon, Lucie Gamon, Amandine Pisoni, Safa Aouinti, Marie‐Christine Picot, Jean Bousquet

**Affiliations:** ^1^ Department of Internal Medicine and Geriatrics MUSE University Montpellier France; ^2^ INSERM U 1058/EFS University Hospital Montpellier France; ^3^ Clinical Research and Epidemiology Unit University Hospital Montpellier France; ^4^ Department of Dermatology and Allergy Universitätsmedizin Berlin Germany

**Keywords:** BNT162b2 vaccine, boost vaccine dose, nursing home residents, SARS‐CoV‐2 spike antibodies

To the Editor,

In France, a primary series of two BNT162b2 COVID‐19 vaccine doses was administered in most nursing home (NH) residents in January‐March 2021, with a third dose 6 months later. Two doses of BNT162b2 provide higher humoral response and protection against SARS‐CoV‐2 in residents with prior COVID‐19 than in those without prior COVID‐19.^1^, ^2^ It is unknown as to whether COVID‐19 occurring after the second dose may improve the efficacy and humoral response of the third vaccine dose in residents.

We compared COVID‐19 incidence 6 months after the third vaccine dose and Immunogobulin G levels against the SARS‐CoV‐2 Receptor‐Binding Domain (RBD)‐IgG antibody levels 3–4 weeks and 6 months after the third dose in residents without any history of COVID‐19 (group 1), those infected before any vaccination (group 2) or infected between the second and third vaccine doses (group 3).

## 1 METHODS

**FIGURE 1:**
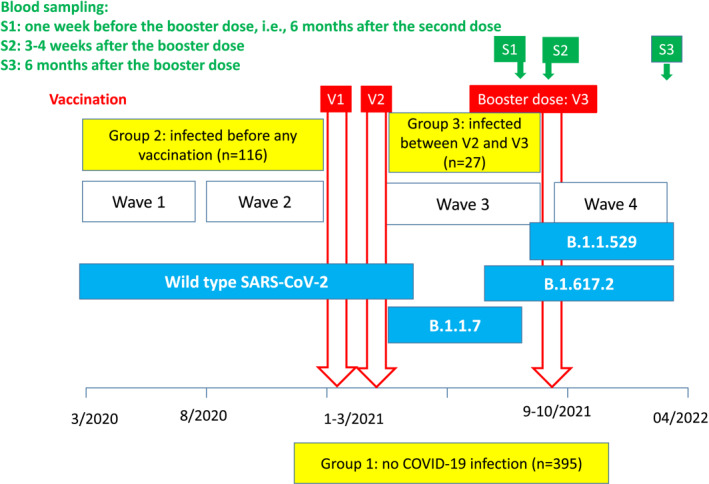
SARS‐CoV‐2 types involved in the different waves of COVID‐19 outbreaks faced by studied nursing home (NH) residents.

Between March 2020 and April 2022, residents from NHs facing COVID‐19 outbreaks (Figure 1) were studied in the CONsort COVID‐19 programme, in accordance with the European Geriatric Medicine Society guidance (online Supplement).^3^, ^4^ As soon as a resident developed COVID‐19 in a NH, all residents and staff members were tested and repeatedly tested every week using quantitative reverse transcription polymerase chain reaction (RT‐PCR) on nasopharyngeal swab until no new cases were diagnosed.^5^ Residents for whom informed consent was obtained were tested for RDB‐IgG (SARS‐CoV‐2 IgG II Quant assay, Abbott Diagnostics) and nucleocapsid protein (N Protein‐IgG) (Abbott Diagnostics) 3–4 weeks after and 6 months after the third dose) (see online Supplement). COVID‐19 was confirmed either by a positive RT‐PCR or by detectable N‐protein IgG. We compared COVID‐19 incidence and moderate to severe COVID‐19 during the 6 months after the third vaccine dose and RBD‐IgG levels 3–4 weeks and 6 months after the third vaccine dose in each of the 3 groups using Wilcoxon–Mann–Whitney 2‐sided tests. The study was approved by the Montpellier University Hospital institutional review board (Institut de recherche en Biothérapie de Montpellier [IRB‐MTP]_2020_06_202000534 and IRB‐MTP _2021_04_202000534).

## 2 RESULTS

Among the 540 residents from 14 NHs (see online supplement), 216 (40%) developed COVID‐19 within 6 months after the third vaccine dose. COVID‐19 incidence was comparable in residents infected between the second and third doses (group 3) and in those without prior SARS‐CoV‐2 (group 1) (44.4% vs. 44.0%, *p* = 0.97) and was lower in residents with COVID‐19 before any vaccination (25.4%, *p* < 0.01) (group 2). Incidence of moderate or severe COVID‐19 was comparable in groups 1 and 3 (*p* = 0.49) and lower in group 2 (<0.01 vs. group 1 and 0.05 vs. group 3) (Table 1). Median RBD IgG levels measured 4–6 weeks and 6 months after the booster dose were comparable in residents of groups 1 and 3 and higher in group 2 (Table 1).

**TABLE 1:** Demographic characteristics, incidence of COVID‐19 within 6 months after the third vaccine dose and RBD IgG 3–4 weeks and 6 months after the third vaccine dose in residents without prior COVID‐19 (group 1), with COVID‐19 before vaccination (group 2) or with COVID‐19 between the second and third vaccine doses (group 3).

	Group 1: No prior COVID‐19 (*n* = 395)	Group 2: COVID‐19 before vaccination (*n* = 118)	Group 3: COVID‐19 between the 2^nd^ and 3^rd^ vaccine doses (*n* = 27)	*p* value
Resident characteristics				
Age, mean (SD), y	88.3 (7.8)	88.5 (7.7)	90.2 (6.2)	0.54
Women/Men, no. (%)	286 (72.4)/109 (27.6)	93 (78.8)/25 (21.2)	22 (81.5)/5 (18.5)	0.26
COVID‐19 within 6 months after 3rd vaccine, no. (%)	174 (44.0)	30 (25.42)	12 (44.4)	0.00125
Asymptomatic	128 (32.4)	24 (20.3)	7 (25.9)	^a^ vs ^b^: 0.0034
Moderate or severe	46 (11.6)^a^	6 (5.1)^b^	5 (18.5)^c^	^a^ vs ^c^: 0.49
				^b^ vs ^c^: 0.05
RBD‐IgG, median (IQRs)^a^, BAU/mL^b^, (number)				
Three to 4 weeks after 3^rd^ vaccine dose	1873 (777–4068)^g^, (*n* = 395)	4107 (2049–7123)^h^, (*n* = 118)	1953 (407–10,034)^i^, (*n* = 27)	^g^ vs ^h^: 0.00000000028
				^g^ vs ^i^: 0.79
				^h^ vs ^i^: 0.08
Six months after 3^rd^ vaccine dose	491 (237–1081)^j^, (*n* = 148)	1924 (729–3687)^k^, (*n* = 68)	932 (443–2761)^I^, (*n* = 19)	^j^ vs ^k^: 0.0000000005
				^j^ vs ^i^: 0.24
				^k^ vs ^i^: 0.08

^a^ BAU/ml: Binding Antibody Units per mL.

^b^ IQR: Interquartile range.

## 3 DISCUSSION

This real‐world study in NH residents shows that in contrast to SARS‐CoV‐2 infection before vaccination, infection occurring between the second and third vaccine doses does not increase protection provided by the third vaccine dose against subsequent infection by the Omicron variant or the humoral response when compared to residents without prior COVID‐19.

Even limited by the small sample size, with possible lack of representativeness, this result suggests that vaccinated residents who become infected by COVID‐19 should be offered the same vaccine schedule as vaccinated residents without COVID‐19 history. Even if residents having recovered from COVID‐19 in the first stages of the epidemic may differ from the general population of NHs in which the death rate was around 30% before vaccines became available,^6^ the results of our study suggest that protection and immune response conferred by the third vaccine dose is higher in residents infected by COVID‐19 prior to vaccination than in other residents.

## AUTHOR CONTRIBUTIONS

Hubert Blain has full access to all the data in the study and takes responsibility for the integrity of the data and the accuracy of the data analysis. *Design and conduct of the study*: Blain, Tuaillon, Bousquet. *Collection, management, analysis, and interpretation of the data*: all authors. *Drafting of the manuscript*: Blain, Tuaillon, Bousquet. *Critical revision of the manuscript*: all authors. *Decision to submit the manuscript for publication*: Blain.

## CONFLICT OF INTEREST STATEMENT

Authors declare no conflicts of interest/Competing interests.

## Supporting information

Supplementary Material
