# Supplementary material for: COVID‐19 after BNT162b2 two‐dose primary series does not improve the efficacy of a booster dose in nursing home residents
Source: Clin Transl Allergy. 2023 Mar 22;13(3):e12224. doi: 10.1002/clt2.12224 (PMC10033849; doi:10.1002/clt2.12224)
Supplement: Supplementary file 1 — Supplementary Material [file CLT2-13-e12224-s001.docx]

Since March 2019, around 6,000 residents from 122 nursing homes are being prospectively followed in the Montpellier area (French Occitanie region). Among the 58 nursing homes having faced a COVID-19 outbreak of wild type SARS-CoV-2 in 2020, a follow-up of post-vaccine antibody response was accepted by residents from 22 of them.^1^ Blood testing was performed to assess RBD-IgG (IgG II Quant assay, Abbott Diagnostics; upper limit: 5,680 BAU) and nucleocapsid-IgG (Abbott Alinity) 3 weeks after the first jab (i.e., just before the second one) and 6 weeks after the second jab.^2^ Among those 22 NHs, 20 accepted to organize measurements of RBD-IgG and nucleocapsid-IgG 1‒3 days before and 21‒28 days after the third dose (Blain-Allergy-2022). Among those 20 NHs, 14 had at least one resident with a positive RT-PCR in 2021 (wild type SARS-CoV-2 between January and February 2021; SARS-CoV-2 B.1.1.7 between February and October 2021; B.1.617.2 between July and October 2021). These 14 NHs agreed to continue to follow the post-vaccine antibody response with measures of RBD-IgG 6 months after the third vaccine dose. Among the 1,101 residents for whom at least one blood measurement has been performed since March 2020, 742 accepted a blood measurement 6 months after the third vaccine dose. Among those 742 residents, we have complete data (demographic characteristics, incidence of COVID-19 within 6 months after the third vaccine dose, RBD IgG 3-4 weeks and 6 months after the third vaccine dose, follow-up to confirm COVID-19 diagnosis: either positive RT-PCR or detection of N-protein IgG appearance since the last measure) for 540 of them. Demographic characteristics of the 540 residents entered in the study were not different from those of the 742 residents. Analyses were performed using the SAS Enterprise Guide, v 7.3 (SAS Institute Inc). The statistical significance threshold was set at 5%.

1. Blain H, Tuaillon E, Gamon L, Pisoni A, Miot S, Rolland Y, Picot MC, Bousquet J. Antibody response after one and two jabs of the BNT162b2 vaccine in nursing home residents: The CONsort-19 study. Allergy. 2022;77(1):271-281. doi: 10.1111/all.15007.
2. Blain H, Tuaillon E, Gamon L, Pisoni A, Miot S, Picot MC. Strong Decay of SARS-CoV-2 Spike Antibodies after 2 BNT162b2 Vaccine Doses and High Antibody Response to a Third Dose in Nursing Home Residents. J Am Med Dir Assoc. 2022;23(5):750-753. doi: 10.1016/j.jamda.2022.02.006.
